# Is Central Sensitisation the Missing Link of Persisting Symptoms after COVID-19 Infection?

**DOI:** 10.3390/jcm10235594

**Published:** 2021-11-28

**Authors:** Lisa Goudman, Ann De Smedt, Marc Noppen, Maarten Moens

**Affiliations:** 1 Department of Neurosurgery, Universitair Ziekenhuis Brussel, Laarbeeklaan 101, 1090 Brussels, Belgium; maarten.moens@uzbrussel.be; 2 STIMULUS Research Group (reSearch and TeachIng neuroModULation Uz bruSsel), Vrije Universiteit Brussel, Laarbeeklaan 103, 1090 Brussels, Belgium; Ann.DeSmedt@uzbrussel.be; 3 Center for Neurosciences (C4N), Vrije Universiteit Brussel, Laarbeeklaan 103, 1090 Brussels, Belgium; 4 Pain in Motion (PAIN) Research Group, Department of Physiotherapy, Human Physiology and Anatomy, Faculty of Physical Education and Physiotherapy, Vrije Universiteit Brussel, Laarbeeklaan 103, 1090 Brussels, Belgium; 5 Research Foundation—Flanders (FWO), 1090 Brussels, Belgium; 6 Department of Physical Medicine and Rehabilitation, Universitair Ziekenhuis Brussel, Laarbeeklaan 101, 1090 Brussels, Belgium; 7 Chief Executive Officer, Universitair Ziekenhuis Brussel, Laarbeeklaan 101, 1090 Brussels, Belgium; marc.noppen@uzbrussel.be; 8 Department of Radiology, Universitair Ziekenhuis Brussel, Laarbeeklaan 101, 1090 Brussels, Belgium

**Keywords:** COVID-19, persisting symptoms, fatigue, nociplastic pain, functional status, central sensitisation

## Abstract

Patients recovered from a COVID-19 infection often report vague symptoms of fatigue or dyspnoea, comparable to the manifestations in patients with central sensitisation. The hypothesis was that central sensitisation could be the underlying common aetiology in both patient populations. This study explored the presence of symptoms of central sensitisation, and the association with functional status and health-related quality of life, in patients post COVID-19 infection. Patients who were previously infected with COVID-19 filled out the Central Sensitisation Inventory (CSI), the Post-COVID-19 Functional Status (PCFS) Scale and the EuroQol with five dimensions, through an online survey. Eventually, 567 persons completed the survey. In total, 29.73% of the persons had a score of <40/100 on the CSI and 70.26% had a score of ≥40/100. Regarding functional status, 7.34% had no functional limitations, 9.13% had negligible functional limitations, 37.30% reported slight functional limitations, 42.86% indicated moderate functional limitations and 3.37% reported severe functional limitations. Based on a one-way ANOVA test, there was a significant effect of PCFS Scale group level on the total CSI score (F(4,486) = 46.17, *p* < 0.001). This survey indicated the presence of symptoms of central sensitisation in more than 70% of patients post COVID-19 infection, suggesting towards the need for patient education and multimodal rehabilitation, to target nociplastic pain.

## 1. Introduction

Currently, the outbreak of the coronavirus disease 2019 (COVID-19) pandemic is still a serious global public health concern. This novel coronavirus, was first discovered in Wuhan, China, in 2019 and afterwards rapidly spread throughout the world, causes a disease that manifested itself with fever, cough, encephalitis, myalgia, fatigue, muscle weakness, arthralgia, anosmia, and impairment in other bodily functions in the acute phase [1,2,3,4,5]. While mild symptoms are reported in approximately 85% of the cases, a substantial proportion of patients with COVID-19 develop acute respiratory distress syndrome (ARDS) and critical illness [6]. Up to 17% of cases needed high-dependency/intensive care unit treatment due to hypoxemic pulmonary failure [7]. Besides the impact on the respiratory system, coronaviruses had an effect on other systems as well including the central nervous system, cardiovascular system, musculoskeletal system, and gastrointestinal system [2,3,8,9,10,11]. As the COVID-19 pandemic continues, signs and symptoms, such as persistent fatigue or depression, which continue or develop after acute COVID-19 are reported, and are denoted as “long COVID” [12].

In patients who are suffering from chronic non-specific pain, central sensitisation (i.e., an amplification of neural signalling within the central nervous system that elicits pain hypersensitivity [13]) often serves as an underlying neurophysiological mechanism to explain the manifestations [14]. Especially, in patients in whom there is an absence of a clear origin of nociceptive input or absence of enough tissue damage to explain the experienced pain, disability, and other symptoms, central nervous system sensitisation is often proposed. Central sensitisation has been denoted as an important contributor or a common aetiology in a variety of chronic musculoskeletal conditions, including fibromyalgia, chronic fatigue syndrome, and irritable bowel syndrome [15]. Despite the lack of a solid outcome measurement, the Central Sensitization Inventory (CSI) was previously introduced as a screening instrument for clinicians to help identify patients with central sensitisation [15]. In post COVID-19 patients, potential long-term secondary effects on the musculoskeletal system such as muscle weakness, decreased muscle mass, and myopathies have been brought to attention [16]. Persisting symptoms are a frequently reported complaint in patients recovered from COVID-19 infection with at least one symptom, particularly fatigue and dyspnoea [17]. Fatigue is also one of the core symptoms in central sensitisation disorders [18], leading to the hypothesis that central sensitisation might be the underlying common aetiology in patients with chronic pain and patients post COVID-19 infection.

The goal of this study was to gain further insight in the presence of central sensitisation as underlying factor for long-term secondary effects in post COVID-19 patients. Additionally, we evaluated whether there was an association between total scores on the CSI and the functional status after COVID-19 infection. Therefore, the aim of this study was to explore the presence of symptoms of central sensitisation, and the association with functional status and health-related quality of life, in patients post COVID-19 infection.

## 2. Materials and Methods

### 2.1. Study Participants

This study used a cross-sectional online survey design with a convenience sample of individuals self-reporting the presence of a post COVID-19 infection state. The survey population comprised all Dutch speaking adults, living in Belgium. The sampling frame consisted of all post COVID-19 patients who were active on social media since the survey was spread on LinkedIn, Facebook and Instagram several times between 4 June 2021 and 22 August 2021. Additionally, personal contacts of the research group members who were infected with COVID-19 were asked to complete the online survey. No specific criteria were imposed regarding the time frame after infection.

On the first page of the survey, all respondents were informed that the survey was completely anonymous and that the information would only be used for this study. Additionally, they were informed about the main goal of this survey. No financial or other incentives were provided. The survey took around 10 min to complete.

The study protocol was approved by the central ethics committee of Universitair Ziekenhuis Brussels (B.U.N. 1432021000484) on 26 May 2021. The study was registered on clinicaltrials.gov (NCT04912778). The study was conducted according to the revised Declaration of Helsinki (1998).

### 2.2. Data Collection

The online survey consisted of three validated questionnaires (in a random order) to evaluate the functional status, health-related quality of life and symptoms of central sensitisation. Additionally, demographics were questioned (age, sex, time of COVID-19 infection (based on symptoms), availability of a test, length, and weight). In the case that respondents underwent a test to confirm a COVID-19 infection, this was denoted as a confirmatory diagnosis. Respondents without the availability of a COVID-19 test result were denoted as a presumptive diagnosis.

Symptoms of central sensitisation were assessed with the Central Sensitization Inventory (CSI). The CSI consists of 25 symptom-related opinions that the patient had to score on a 5-point Likert scale [15]. A total score of ≥40/100 indicated the presence of central sensitisation (sensitivity: 81%, specificity: 75%) [15]. The CSI has good clinimetric properties for assessing symptoms of central sensitisation and is validated in Dutch [19,20]. Additionally, respondents were categorised based on central sensitisation-related severity into three subgroups: (i) low level, (ii) medium level, or (iii) high level of central sensitisation-related symptom severity using the freely accessible online calculator (https://www.pridedallas.com/questionnaires, accessed on 19 November 2021) [21].

The post COVID-19 functional status was evaluated by the Post-COVID-19 Functional Status (PCFS) Scale, using the self-reporting version of this questionnaire [22,23]. This intuitive scale is ordinal, with 6 steps ranging from grade 0 (no functional limitations) to grade 4 (severe functional limitations) and grade 5 (death), and covers the entire range of functional outcomes by focusing on limitations in usual duties/activities either at home or at work/study, as well as changes in lifestyle.

The EuroQol with five dimensions and three levels (EQ5D-3L) [24] is a standardised health-related quality of life questionnaire to provide a generic measure of health for clinical and economic appraisal [25]. The EQ5D-3L consists of a descriptive system and a visual analogue scale (VAS). The descriptive system contains five dimensions (mobility, self-care, usual activities, pain/discomfort, anxiety/depression). Each dimension has three response levels. In the second part of the questionnaire, a standard vertical 20 cm VAS was implemented to record an individual’s rating for their current health-related quality of life state. The responses to the EQ-5D dimensions were converted into a single index value for all health states [26]. Health state index scores generally range from less than zero (where zero is a health state equivalent to death; negative values are valued as worse than death) to one (perfect health), with higher scores indicating a higher health utility.

### 2.3. Statistical Analysis

Survey data were collected through LimeSurvey. All analyses were performed in R Studio version 1.4.1106 (R version 4.0.5). *P*-values of 0.05 or less were considered statistically significant. Descriptive statistics were provided as means with corresponding standard deviation (SD). Two-sample *t*-tests were performed to evaluate the effect of sex and the presence of a test on CSI, EQ5D and EQ5D VAS scores. Pearson correlation coefficients were calculated between total CSI scores, EQ5D scores and EQ5D VAS scores on the one hand and time since infection and body mass index (BMI) on the other hand. A point-biserial correlation was calculated between PCFS Scale scores and CSI total scores and between PCFS Scale scores and EQ5D scores. One-way ANOVA testing was used to explore the effect of PCFS Scale scores on CSI total scores and EQ5D scores, with corresponding Tukey HSD test for post hoc comparisons. All analyses were performed on data as observed, meaning that for respondents with incomplete data, all data that were available was used.

## 3. Results

### 3.1. Demographic Statistics

In total, 741 respondents who were previously infected with COVID-19 opened the survey between 4 June 2021 and 30 August 2021. Of those 741 respondents, 567 started to complete the survey. Demographics were available for 567 respondents; the CSI was filled in by 491 persons, the EQ5D-3L by 547 respondents, the EQ5D VAS by 537 persons and the PCFS Scale by 504 persons. Seventy-seven (13.58%) males and 490 (86.42%) females completed the survey. Respondents had a mean age of 46.5 (SD: 11.4) years and a BMI of 26.5 (SD: 5.42) kg/m^2^. Respondents were infected with COVID-19 between 22 January 2020 and 25 July 2021. The mean time between the infection and the time of completing this survey was 287 days (SD: 150).

### 3.2. Symptoms of Central Sensitisation, Functional Status and Health-Related Quality of Life

The mean score on the CSI was 45.9 (SD: 13.1), where 146 (29.73%) of the persons had a score of <40/100 on the CSI and 345 (70.26%) of a score ≥40/100. In total, 21 respondents (4.28%) could be classified with a low level of central sensitisation-related symptom severity, 152 (30.96%) with a medium level and 318 (64.76%) with a high level of central sensitisation-related symptom severity. Table 1 presents CSI scores for the full sample, separated by sex, the presence of a Covid test and PCFS Scale score. There was a significant difference in CSI score between males (41.2 (SD 13.8)) and females (46.6 (SD 12.8), *t*(86.54) = −3.01, *p* = 0.003), but not between respondents with a confirmatory or presumptive COVID-19 diagnosis (*t*(163.1) = 1.35, *p* = 0.18). There was a positive correlation (r = 0.09, 95% CI from 0.003 to 0.18) between BMI and the total CSI score (*p* = 0.04). Additionally, a positive significant correlation was revealed between total CSI score and time since infection (r = 0.14, 95% CI from 0.05 to 0.23, *p* = 0.002).

**Figure 1:**
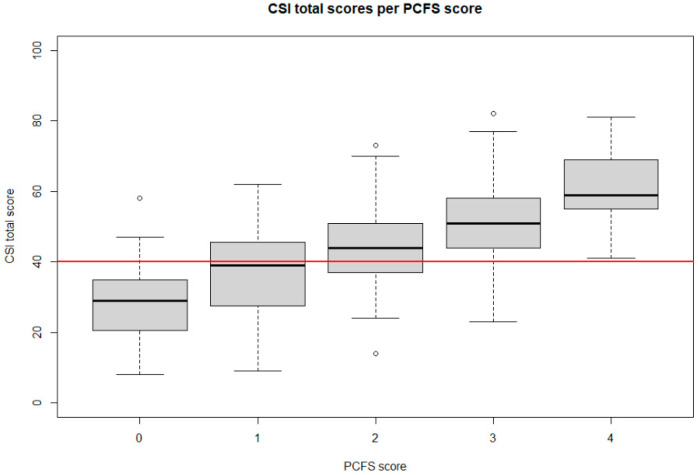
Boxplot of total score on the CSI by PCFS Scale score for all respondents. The red line is presents the cut-off value of the CSI at 40/100 to denote a person as having symptoms of central sensitisation. Abbreviations. CSI: Central Sensitization Inventory, PCFS: Post-COVID-19 Functional Status Scale.

Concerning the PCFS, 37 persons (7.34%) had no functional limitations (grade 0), 46 (9.13%) had negligible functional limitations (grade 1), 188 (37.30%) reported slight functional limitations (grade 2), 216 (42.86%) indicated moderate functional limitations (grade 3) and 17 (3.37%) reported severe functional limitations (grade 4). Figure 1 presents the CSI scores for each level of the PCFS Scale.

The mean EQ5D-3L index score was 0.57 (SD: 0.23) and the EQ5D VAS mean score was 56.6 (SD: 18.2) (Table 1). For the mobility component of the EQ5D-3L, 56.96% of the respondents had no problems, 40.36% had some problems with mobility and 2.68% was confined to bed. For the self-care component, 86.98% reported no problems, 12.30% some problems and 0.72% was unable to wash or dress himself/herself. For usual activities, 17.67% had no problems, 62.66% some problems and 19.67% was unable to perform usual activities. No pain or discomfort was reported by 10.42%, moderate pain or discomfort by 74.59% and extreme pain or discomfort by 14.99%. For anxiety/depression, 57.40% indicated not being anxious or depressed, 38.39% was moderately anxious or depressed and 4.20% was extremely anxious or depressed. There were no statistically significant correlations between EQ5D or EQ5D VAS scores and BMI or time since infection. There was a significant difference in EQ5D VAS scores between males (61.8 (SD 18.6)) and females (55.8 (SD 18.1), *t*(96.51) = 2.56, *p* = 0.01), but not between respondents with a confirmatory or presumptive COVID-19 diagnosis (*t*(163.5) = 0.20, *p* = 0.84). No significant differences in EQ5D scores were found for sex (*t*(100.1) = 1.14, *p* = 0.26) or COVID-19 diagnosis (*t*(185.2) = −0.03, *p* = 0.98).

### 3.3. Association between Symptoms of Central Sensitisation, Functional Status and Health-Related Quality of Life

**Figure 2:**
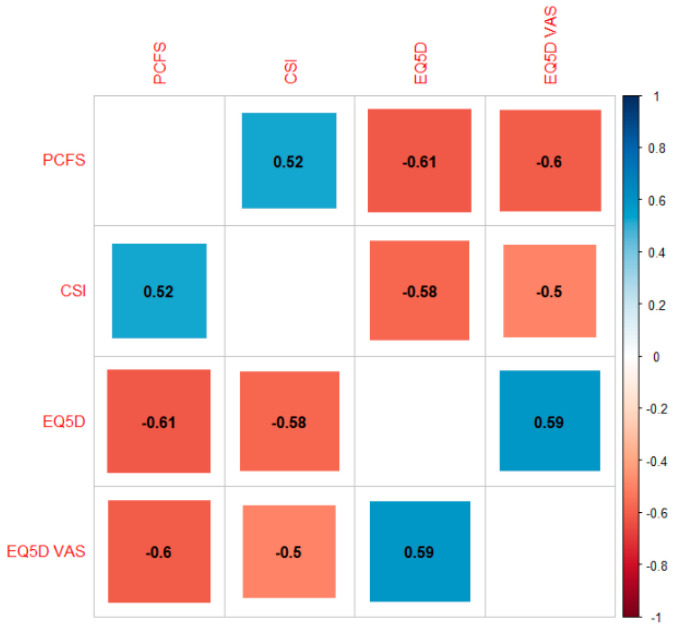
Correlation plot. Correlation coefficients range from −1 (red) to +1 (blue) and presented with the actual value on the plot. Abbreviations. CSI: Central Sensitization Inventory, PCFS: Post-COVID-19 Functional Status Scale, VAS: Visual Analogue Scale.

A significant positive correlation was revealed between PCFS Scale scores and CSI scores (r = 0.52, 95% CI from 0.45 to 0.58, *p* < 0.001). Statistically significant negative correlations were revealed between CSI scores and EQ5D scores (r = −0.58, 95% CI from −0.51 to −0.63, *p* < 0.001), CSI scores and EQ5D VAS scores (r = −0.50, 95% CI from −0.43 to −0.56, *p* < 0.001) and PCFS Scale scores and EQ5D scores (r = −0.61, 95% CI from −0.56 to −0.67, *p* < 0.001) (Figure 2).

**Figure 3:**
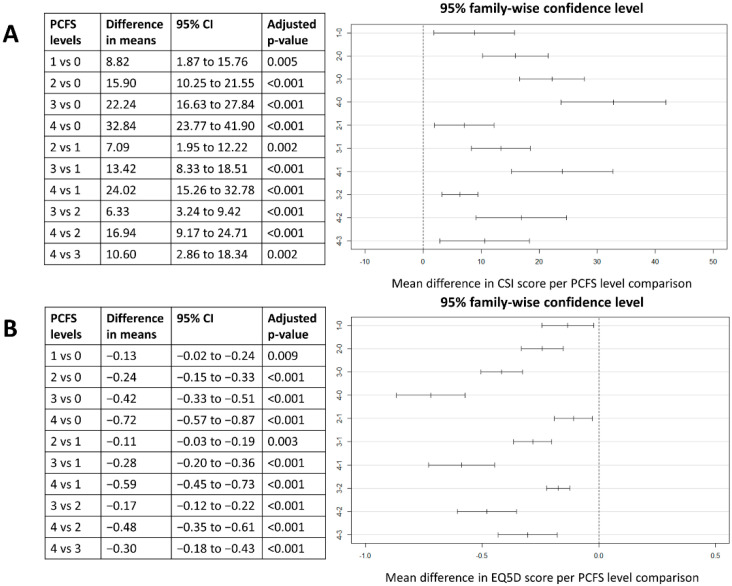
Post hoc comparisons between CSI scores (**A**) and EQ5D scores (**B**) for the different PCFS Scale group levels with a visual presentation of the 95% family-wise confidence intervals for all group level comparisons. Abbreviations. CI: confidence interval, CSI: Central Sensitisation Inventory, PCFS: Post-COVID-19 Functional Status Scale, vs: versus.

Based on a One-way ANOVA testing, there was a significant effect of PCFS group level on the total CSI score at the 5% level (F(4,486) = 46.17, *p* < 0.001). Post hoc comparison using the Tukey HSD test indicated that mean score for the PCFS group 0 was significantly lower than for PCFS 1 group (mean difference of 8.82 (95% CI from 1.87 to 15.76), *p* = 0.005). Additionally, the mean difference in CSI score between PCFS groups 1 and 2 (mean difference of 7.09 (95% CI from 1.95 to 12.22), *p* = 0.002), groups 2 and 3 (mean difference of 6.33 (95% CI from 3.24 to 9.42), *p* < 0.001), and groups 3 and 4 (mean difference of 10.60 (95% CI from 2.86 to 18.34), *p* = 0.002) were statistically significant. Similarly, there was a significant effect of PCFS group level on the EQ5D score at the 5% (F(4,499) = 83.41, *p* < 0.001). Post hoc comparison using the Tukey HSD test indicated a statistically significant difference in EQ5D scores for all level comparisons of the PCFS Scale. All post hoc tests can be found in Figure 3.

## 4. Discussion

In 2017, the International Association for the Study of Pain (IASP) introduced the term nociplastic pain as mechanistic pain descriptor in addition to nociceptive and neuropathic pain [27]. The underlying mechanism of nociplastic pain is central sensitisation, whereby several dysfunctions within the central nervous system, including altered sensory processing in the brain, altered activity in brain-orchestrated nociceptive facilitatory pathways, and poor functioning of endogenous analgesia, can lead to an increased responsiveness to a variety of sensory inputs and/or hypersensitivity to external stimuli such as light, sound or chemical substances [14]. The aim of this study was to evaluate whether there were indications for symptoms of central sensitisation to explain persisting symptoms in patients post COVID-19 infection. Based on the CSI, a screening questionnaire to identify the presence of symptoms of central sensitisation, 70.26% of the respondents reported total CSI scores of ≥40/100, indicating the presence of central sensitisation in this population. Furthermore, when classifying patients according to the level of central sensitisation-related symptom severity, 4.28% were classified as low level, 30.96% as medium level and 64.76% as high level. Therefore, this online survey is suggestive for the presence of symptoms of central sensitisation in post COVID-19 patients. Mean values of 45.9 (SD: 13.1) on the CSI were revealed in this population, which was in line with values obtained in patients with chronic low back pain (total score of 41.6 (SD: 14.8)) [19] and a large group of 368 chronic pain patients (total score of 43.88 (SD: 17.67)) [20]. In healthy persons, mean values of 21.55 (SD: 10.92) [20] and 28.9 (SD: 13.5) [19] were reported in datasets of 49 and 40 persons, respectively. These reference data were observed in studies with other designs; therefore, they only provide a rough indication to better interpret the currently observed findings in post COVID-19 patients. Future studies could further explore the suggestion of central sensitisation as an underlying mechanism of persisting symptoms in post COVID-19 patients by a thorough evaluation of the underlying pain state. Quantitative sensory testing, i.e., a widely used method to measure patients’ verbal and behavioural response to quantifiable sensory stimuli, may be used to assess detection and/or pain thresholds, temporal summation (as measure of imbalanced pain facilitation by controlling for increasing evoked pain by fixed repetitive stimuli) and conditioned pain modulation (as measure of imbalanced pain inhibition by controlling for the ability to reduce evoked pain by a second stimulus) [28], while offset analgesia and functional neuroimaging could evaluate cerebral pain processing [29]. In line with previous research in patients with chronic pain, less efficacious conditioned pain modulation, as an indirect measure for the functioning and efficiency of the endogenous descending nociceptive inhibitory systems in humans [30,31,32] (presumably due to a shift between nociceptive facilitation and nociceptive inhibition), increased nociceptive facilitation [33] and decreased pain thresholds [34,35] are expected if the stated hypothesis based on this online survey is valid.

The exact underlying pathogenetic mechanisms of central sensitisation are not yet fully unravelled; however, it is suggested that infectious agents play a role in, for example myalgic encephalomyelitis/chronic fatigue syndrome (ME/CFS) [36]. It was previously proposed that several viruses (Epstein-Barr virus [37,38], cytomegalovirus [39], human herpesvirus 6-8 [40,41,42] or bacteria such as mycoplasma [43]) trigger ME/CFS and may reactivate under various conditions, thereby inducing inflammation and immune dysregulation [36]. In comparison to healthy controls, patients who underwent a COVID-19 infection presented with dysregulated immune response (decreased T, B and NK cells and increased inflammatory cytokines) [44], indicating that immune dysfunction plays critical roles in disease progression [45]. Additionally, viral infections also lead to neuroinflammation with the activation of microglia and astrocytes, which leads to the release of proinflammatory cytokines and chemokines [46]. Central cytokines and chemokines play a role in inducing hyperalgesia and allodynia, while a sustained increase leads to chronic widespread pain at several body locations, suggesting that neuroinflammation drives widespread chronic pain through central sensitisation [47]. Therefore, neuroinflammation is expected to play a role in persisting symptoms after COVID-19 infection as well [48,49].

In this survey, it was also revealed that the degree of functional limitations was significantly associated with the degree of symptoms of central sensitisation. The PCFS Scale measured the impact of symptoms on the functional status of patients after COVID-19 infection, whereby previous research already revealed a gradual increase in impairment in work and usual activities and the intensity of symptoms from grade 2 to 4 on the PCFS Scale [50]. The current study demonstrated gradual increases in symptoms of central sensitisation in all grades (except death) of the PCFS Scale, ranging from no symptoms up to severe functional limitations. Thus, it seems that patient-reported consequences of COVID-19 and their effect on functional status are associated with symptoms of central sensitisation. Furthermore, statistically significant negative correlations were found between PCFS Scale score and EQ5D utility and VAS scores, indicating that a higher impact of symptoms on the functional status of patients after a COVID-19 infection was associated with lower health-related index scores and lower health-related quality of life. This correlation has also been reported in patients with lumbar degenerative disorders, whereby the authors evaluated whether the EQ5D score could be derived from other currently used questionnaires with negative (i.e., not accurate) results [51]. Therefore, it might be hypothesised that despite the association between the PCFS Scale and health-related quality of life in patients post COVID-19 infection, both questionnaires are evaluating different aspects and are not interchangeable. The PCFS Scale could be used at the time of hospital discharge, and to monitor functional status after hospital discharge [50], which could potentially help clinicians to determine an appropriate treatment strategy at the early stages after COVID-19 infection [52]. Nevertheless, this only provides an indication about the functional status of these patients, and not the health-related quality of life.

One of the limitations of this online survey is the unequal sex distribution with a higher response rate of females compared to males. Male patients have higher odds of requiring intensive treatment unit admission and higher odds of death compared to females, although there is no difference in the proportion of males and females with confirmed COVID-19 [53]. Nevertheless, it appeared that female post COVID-19 patients were more likely to respond to this online survey than males. Additionally, patients who were associated with a patient support group on social media and who were interested in post COVID sequelae were more likely to receive the link to the survey, which may have caused a selection bias. Moreover, the survey was only spread in the Flemish speaking part of Belgium. Therefore, the results cannot be generalised towards all patients who underwent a COVID-19 infection. Finally, in this survey, only one evaluation took place, namely after infection with COVID-19. Other study designs with a longitudinal aspect (for example a cohort study in 1000 respondents without symptoms) in which evaluations are performed at several time points could have provided information about causality. More information on existing comorbidities, medication use, the presence of structural dysfunctions, psychological factors or severity of COVID-19 infection in addition to pre-infection status should be recorded in future studies to evaluate their potential influence on the relation between symptoms of central sensitisation and COVID-19 infection.

## 5. Conclusions

This online survey indicated the presence of symptoms of central sensitisation in a sample of post COVID-19 infection patients. Moreover, the more functional limitations due to COVID-19 infection, the higher the degree of symptoms of central sensitisation. The results of this study suggest the need for multimodal rehabilitation and patient education in patients after COVID-19 infection.

## Figures and Tables

**Table 1:** Total scores on the CSI, separated for categorical variables. Abbreviations. N: number of respondents; PCFS: Post-COVID-19 Functional Status Scale, SD: standard deviation.

Variable	Level	Mean CSI Score	Mean EQ5D	Mean EQ5D Vas
Sample		45.9 (SD 13.1) (*N* = 491)	0.57 (SD 0.23) (*N* = 547)	56.6 (SD 18.2) (*N* = 537)
Sex	Male	41.2 (SD 13.8) (*N* = 68)	0.60 (SD 0.23) (*N* = 75)	61.8 (SD 18.6) (*N* = 74)
	Female	46.6 (SD 12.8) (*N* = 423)	0.56 (SD 0.23) (*N* = 472)	55.8 (SD 18.1) (*N* = 463)
COVID-19 DIAGNOSIS	Confirmatory	45.5 (SD 13.2) (*N* = 390)	0.57 (SD 0.24) (*N* = 433)	56.6 (SD 17.9) (*N* = 112)
	Presumptive	47.4 (SD 12.5) (*N* = 101)	0.57 (SD 0.22) (*N* = 114)	57.0 (SD 19.5) (*N* = 425)
PCFS	Score 0	28.5 (SD 11.8) (*N* = 35)	0.87 (SD 0.15) (*N* = 37)	78.4 (SD 15.8) (*N* = 37)
	Score 1	37.3 (SD 13.1) (*N* = 44)	0.73 (SD 0.12) (*N* = 46)	72.3 (SD 12.7) (*N* = 46)
	Score 2	44.4 (SD 10.8) (*N* = 187)	0.63 (SD 0.15) (*N* = 188)	60.7 (SD 13.1) (*N* = 188)
	Score 3	50.7 (SD 11.1) (*N* = 208)	0.45 (SD 0.22) (*N* = 216)	47.3 (SD 15.2) (*N* = 216)
	Score 4	61.3 (SD 10.9) (*N* = 17)	0.15 (SD 0.11) (*N* = 17)	34.1 (SD 14.5) (*N* = 17)

## Data Availability

The data presented in this study are available on motivated request from the corresponding author.

## References

[1] Eliezer M., Eloit C., Hautefort C. (2020). Olfactory Loss of Function as a Possible Symptom of COVID-19—Reply. JAMA Otolaryngol. Head Neck Surg..

[2] Joob B., Wiwanitkit V. (2020). Arthralgia as an initial presentation of COVID-19: Observation. Rheumatol. Int..

[3] Zhao H., Shen D., Zhou H., Liu J., Chen S. (2020). Guillain-Barre syndrome associated with SARS-CoV-2 infection: Causality or coincidence?. Lancet Neurol..

[4] Sun P., Lu X., Xu C., Sun W., Pan B. (2020). Understanding of COVID-19 based on current evidence. J. Med. Virol..

[5] Abdullahi A., Candan S.A., Abba M.A., Bello A.H., Alshehri M.A., Afamefuna Victor E., Umar N.A., Kundakci B. (2020). Neurological and Musculoskeletal Features of COVID-19: A Systematic Review and Meta-Analysis. Front. Neurol..

[6] Pfortmueller C.A., Spinetti T., Urman R.D., Luedi M.M., Schefold J.C. (2021). COVID-19-associated acute respiratory distress syndrome (CARDS): Current knowledge on pathophysiology and ICU treatment—A narrative review. Best Pract. Res. Clin. Anaesthesiol..

[7] Docherty A.B., Harrison E.M., Green C.A., Hardwick H.E., Pius R., Norman L., Holden K.A., Read J.M., Dondelinger F., Carson G. (2020). Features of 20 133 UK patients in hospital with COVID-19 using the ISARIC WHO Clinical Characterisation Protocol: Prospective observational cohort study. BMJ.

[8] Bansal M. (2020). Cardiovascular disease and COVID-19. Diabetes Metab. Syndr..

[9] Cheung K.S., Hung I.F.N., Chan P.P.Y., Lung K.C., Tso E., Liu R., Ng Y.Y., Chu M.Y., Chung T.W.H., Tam A.R. (2020). Gastrointestinal Manifestations of SARS-CoV-2 Infection and Virus Load in Fecal Samples from a Hong Kong Cohort: Systematic Review and Meta-analysis. Gastroenterology.

[10] Zhou Y., Li W., Wang D., Mao L., Jin H., Li Y., Hong C., Chen S., Chang J., He Q. (2020). Clinical time course of COVID-19, its neurological manifestation and some thoughts on its management. Stroke Vasc. Neurol..

[11] Mao L., Jin H., Wang M., Hu Y., Chen S., He Q., Chang J., Hong C., Zhou Y., Wang D. (2020). Neurologic Manifestations of Hospitalized Patients with Coronavirus Disease 2019 in Wuhan, China. JAMA Neurol..

[12] The L. (2021). Understanding long COVID: A modern medical challenge. Lancet.

[13] Woolf C.J. (2011). Central sensitization: Implications for the diagnosis and treatment of pain. Pain.

[14] Nijs J., Lahousse A., Kapreli E., Bilika P., Saracoglu I., Malfliet A., Coppieters I., De Baets L., Leysen L., Roose E. (2021). Nociplastic Pain Criteria or Recognition of Central Sensitization? Pain Phenotyping in the Past, Present and Future. J. Clin. Med..

[15] Neblett R., Cohen H., Choi Y., Hartzell M.M., Williams M., Mayer T.G., Gatchel R.J. (2013). The Central Sensitization Inventory (CSI): Establishing clinically significant values for identifying central sensitivity syndromes in an outpatient chronic pain sample. J. Pain Off. J. Am. Pain Soc..

[16] Candan S.A., Elibol N., Abdullahi A. (2020). Consideration of prevention and management of long-term consequences of post-acute respiratory distress syndrome in patients with COVID-19. Physiother. Theory Pract..

[17] Carfi A., Bernabei R., Landi F. (2020). Persistent Symptoms in Patients after Acute COVID-19. JAMA.

[18] Aaron L.A., Buchwald D. (2001). A review of the evidence for overlap among unexplained clinical conditions. Ann. Intern. Med..

[19] Mayer T.G., Neblett R., Cohen H., Howard K.J., Choi Y.H., Williams M.J., Perez Y., Gatchel R.J. (2012). The development and psychometric validation of the central sensitization inventory. Pain Pract..

[20] Kregel J., Vuijk P.J., Descheemaeker F., Keizer D., van der Noord R., Nijs J., Cagnie B., Meeus M., van Wilgen P. (2016). The Dutch Central Sensitization Inventory (CSI): Factor Analysis, Discriminative Power, and Test-Retest Reliability. Clin. J. Pain.

[21] Cuesta-Vargas A.I., Neblett R., Nijs J., Chiarotto A., Kregel J., van Wilgen C.P., Pitance L., Knezevic A., Gatchel R.J., Mayer T.G. (2020). Establishing Central Sensitization-Related Symptom Severity Subgroups: A Multicountry Study Using the Central Sensitization Inventory. Pain Med..

[22] Corsi G., Nava S., Barco S. (2020). A novel tool to monitor the individual functional status after COVID-19: The Post-COVID-19 Functional Status (PCFS) scale. G. Ital. Cardiol..

[23] Klok F.A., Boon G., Barco S., Endres M., Geelhoed J.J.M., Knauss S., Rezek S.A., Spruit M.A., Vehreschild J., Siegerink B. (2020). The Post-COVID-19 Functional Status scale: A tool to measure functional status over time after COVID-19. Eur. Respir. J..

[24] Rabin R., de Charro F. (2001). EQ-5D: A measure of health status from the EuroQol Group. Ann. Med..

[25] The EuroQol Group (1990). EuroQol—A new facility for the measurement of health-related quality of life. Health Policy.

[26] EuroQol Group (2014). Self-Reported Population Health: An International Perspective Based on EQ-5D.

[27] Aydede M., Shriver A. (2018). Recently introduced definition of “nociplastic pain” by the International Association for the Study of Pain needs better formulation. Pain.

[28] Fillingim R.B., Loeser J.D., Baron R., Edwards R.R. (2016). Assessment of Chronic Pain: Domains, Methods, and Mechanisms. J. Pain Off. J. Am. Pain Soc..

[29] Kosek E., Clauw D., Nijs J., Baron R., Gilron I., Harris R.E., Mico J.A., Rice A.S., Sterling M. (2021). Chronic nociplastic pain affecting the musculoskeletal system: Clinical criteria and grading system. Pain.

[30] Coppieters I., De Pauw R., Kregel J., Malfliet A., Goubert D., Lenoir D., Cagnie B., Meeus M. (2017). Differences between Women with Traumatic and Idiopathic Chronic Neck Pain and Women without Neck Pain: Interrelationships among Disability, Cognitive Deficits, and Central Sensitization. Phys. Ther..

[31] Staud R. (2012). Abnormal endogenous pain modulation is a shared characteristic of many chronic pain conditions. Expert Rev. Neurother..

[32] Kennedy D.L., Kemp H.I., Ridout D., Yarnitsky D., Rice A.S. (2016). Reliability of conditioned pain modulation: A systematic review. Pain.

[33] Soon B., Vicenzino B., Schmid A.B., Coppieters M.W. (2017). Facilitatory and inhibitory pain mechanisms are altered in patients with carpal tunnel syndrome. PLoS ONE.

[34] Imamura M., Chen J., Matsubayashi S.R., Targino R.A., Alfieri F.M., Bueno D.K., Hsing W.T. (2013). Changes in pressure pain threshold in patients with chronic nonspecific low back pain. Spine.

[35] Goubert D., Danneels L., Graven-Nielsen T., Descheemaeker F., Meeus M. (2017). Differences in Pain Processing between Patients with Chronic Low Back Pain, Recurrent Low Back Pain, and Fibromyalgia. Pain Physician.

[36] Rasa S., Nora-Krukle Z., Henning N., Eliassen E., Shikova E., Harrer T., Scheibenbogen C., Murovska M., Prusty B.K., European Network on ME/CFS (EUROMENE) (2018). Chronic viral infections in myalgic encephalomyelitis/chronic fatigue syndrome (ME/CFS). J. Transl. Med..

[37] Straus S.E., Tosato G., Armstrong G., Lawley T., Preble O.T., Henle W., Davey R., Pearson G., Epstein J., Brus I. (1985). Persisting illness and fatigue in adults with evidence of Epstein-Barr virus infection. Ann. Intern. Med..

[38] Holmes G.P., Kaplan J.E., Stewart J.A., Hunt B., Pinsky P.F., Schonberger L.B. (1987). A cluster of patients with a chronic mononucleosis-like syndrome. Is Epstein-Barr virus the cause?. JAMA.

[39] Martin W.J. (1997). Detection of RNA sequences in cultures of a stealth virus isolated from the cerebrospinal fluid of a health care worker with chronic fatigue syndrome. Case report. Pathobiology.

[40] Buchwald D., Cheney P.R., Peterson D.L., Henry B., Wormsley S.B., Geiger A., Ablashi D.V., Salahuddin S.Z., Saxinger C., Biddle R. (1992). A chronic illness characterized by fatigue, neurologic and immunologic disorders, and active human herpesvirus type 6 infection. Ann. Intern. Med..

[41] Yalcin S., Kuratsune H., Yamaguchi K., Kitani T., Yamanishi K. (1994). Prevalence of human herpesvirus 6 variants A and B in patients with chronic fatigue syndrome. Microbiol. Immunol..

[42] Ablashi D.V., Eastman H.B., Owen C.B., Roman M.M., Friedman J., Zabriskie J.B., Peterson D.L., Pearson G.R., Whitman J.E. (2000). Frequent HHV-6 reactivation in multiple sclerosis (MS) and chronic fatigue syndrome (CFS) patients. J. Clin. Virol..

[43] Nasralla M., Haier J., Nicolson G.L. (1999). Multiple mycoplasmal infections detected in blood of patients with chronic fatigue syndrome and/or fibromyalgia syndrome. Eur. J. Clin. Microbiol. Infect. Dis..

[44] Song C.-Y., Xu J., He J.-Q., Lu Y.-Q. (2020). Immune dysfunction following COVID-19, especially in severe patients. Sci. Rep..

[45] Rendeiro A.F., Casano J., Vorkas C.K., Singh H., Morales A., DeSimone R.A., Ellsworth G.B., Soave R., Kapadia S.N., Saito K. (2021). Profiling of immune dysfunction in COVID-19 patients allows early prediction of disease progression. Life Sci. Alliance.

[46] Li T., Chen X., Zhang C., Zhang Y., Yao W. (2019). An update on reactive astrocytes in chronic pain. J. Neuroinflamm..

[47] Ji R.R., Nackley A., Huh Y., Terrando N., Maixner W. (2018). Neuroinflammation and Central Sensitization in Chronic and Widespread Pain. Anesthesiology.

[48] Klein R., Soung A., Sissoko C., Nordvig A., Canoll P., Mariani M., Jiang X., Bricker T., Goldman J., Rosoklija G. (2021). COVID-19 induces neuroinflammation and loss of hippocampal neurogenesis. Res. Sq..

[49] Chowdhury B., Sharma A., Satarker S., Mudgal J., Nampoothiri M. (2021). Dialogue between Neuroinflammation and Neurodegenerative Diseases in COVID-19. J. Environ. Pathol. Toxicol. Oncol..

[50] Machado F.V.C., Meys R., Delbressine J.M., Vaes A.W., Goertz Y.M.J., van Herck M., Houben-Wilke S., Boon G., Barco S., Burtin C. (2021). Construct validity of the Post-COVID-19 Functional Status Scale in adult subjects with COVID-19. Health Qual. Life Outcomes.

[51] Carreon L.Y., Bratcher K.R., Das N., Nienhuis J.B., Glassman S.D. (2014). Estimating EQ-5D values from the Oswestry Disability Index and numeric rating scales for back and leg pain. Spine.

[52] Pant P., Joshi A., Basnet B., Shrestha B.M., Bista N.R., Bam N., Das S.K. (2021). Prevalence of Functional Limitation in COVID-19 Recovered Patients Using the Post COVID-19 Functional Status Scale. JNMA J. Nepal Med. Assoc..

[53] Peckham H., de Gruijter N.M., Raine C., Radziszewska A., Ciurtin C., Wedderburn L.R., Rosser E.C., Webb K., Deakin C.T. (2020). Male sex identified by global COVID-19 meta-analysis as a risk factor for death and ITU admission. Nat. Commun..

